# Creatine consumption and liver disease manifestations in individuals aged 12 years and over

**DOI:** 10.1002/fsn3.3151

**Published:** 2022-11-18

**Authors:** Nikola Todorovic, Darinka Korovljev, Valdemar Stajer, Jagoda Jorga, Sergej M. Ostojic

**Affiliations:** ^1^ Applied Bioenergetics Lab, Faculty of Sport and Physical Education University of Novi Sad Serbia; ^2^ Department of Hygiene and Medical Ecology, School of Medicine University of Belgrade Serbia; ^3^ Department of Nutrition and Public Health University of Agder Kristiansand Norway

**Keywords:** blood liver panel, creatine, diet, hepatic steatosis, liver fibrosis, NHANES

## Abstract

Despite the overwhelming safety evidence concerning creatine intake in various settings, there is still incomplete information whether dietary creatine affects liver health at the population level. The main aim of this cross‐sectional population‐based study was to evaluate the association between creatine intake through regular diet and liver disease manifestations, including liver fibrosis and hepatic steatosis, among individuals aged 12 years and over, using open‐source data from the 2017–2018 U.S. National Health and Nutrition Examination Survey (NHANES). A total of 9254 male and female participants of all ages were included in the 2017–2018 NHANES round. We extracted data from the total sample population for participants who provided dietary data for individual foods via dietary interviews and examination data from liver ultrasound transient elastography. The final study sample consisted of 5957 participants (mean age 44.7 ± 21.0 years; 50.1% women), and the mean dietary creatine intake across the study population was 0.88 ± 0.71 g/day. Liver fibrosis and cirrhosis were diagnosed in 1703 (28.7%) and 288 (4.8%) participants, respectively; hepatic steatosis was identified in 2595 (43.7%) individuals. Binary logistic regression with multivariable model adjusted for age, gender, family income to poverty ratio, body mass index, total energy intake, and alcohol consumption showed that consuming more creatine (≥2 g/day) did not significantly increase the risk of liver fibrosis (OR = 0.92, 95% CI 0.70–1.21, *p* = .57), cirrhosis (OR = 0.94, 95% CI 0.53–1.65, *p* = .82), or hepatic steatosis (OR = 0.77, 95% CI 0.59–1.02, *p* = .07), as compared to participants who ingested <1 g of creatine daily. Dietary exposure to creatine through a regular diet is not associated with an increase in disease manifestations in individuals 12 years and over; further research is warranted to address the effects of excessive creatine intake (≥5 g/day) through a regular diet on liver health at the population level.

## Graphical abstract

The main aim of this cross‐sectional population‐based study was to evaluate the association between dietary creatine intake and liver disease manifestations, including liver fibrosis and hepatic steatosis, among individuals aged 12 and over, using open‐source data from the 2017‐2018 U.S. National Health and Nutrition Examination Survey (NHANES). The 2017‐2018 NHANES round included 9,254 males and females aged 0 to 150. Dietary exposure to additional creatine through a regular diet is not associated with more liver disease manifestations in individuals 12 years and over; further research is warranted to address the effects of excessive creatine intake (≥ 5 grams per day) through a regular diet on liver health at the populational level.

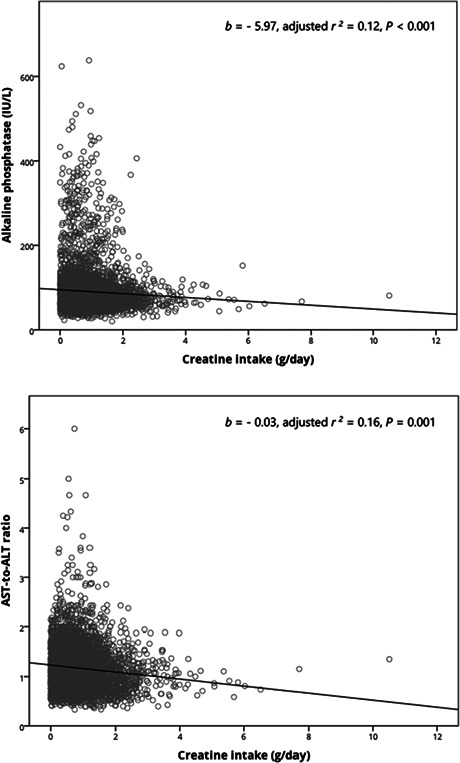

## 1 INTRODUCTION

Creatine is a nutritional compound that plays a significant role in several pathways across the human body. It acts as an intracellular facilitator of high‐energy phosphate metabolism, neuroprotective agent, and immunomodulator (Riesberg et al., 2016), with the liver discerned as a central organ in creatine metabolism (Wyss & Kaddurah‐Daouk, 2000). A diet containing animal products (e.g., milk, red meat, seafood) can account for ~50% of daily creatine requirements, while the other 50% is produced endogenously from amino acids glycine, arginine, and methionine (Brosnan et al., 2011). Humans have to obtain enough creatine from the diet in certain circumstances, which nominates creatine as a conditionally essential nutrient (Ostojic & Forbes, 2022). Dietary creatine appears to be generally safe (Kreider et al., 2022), although excessive intake might cause various digestive issues. In particular, several preclinical studies and case reports suggest that exogenous creatine can adversely affect liver function by elevating liver enzymes on blood tests (Souza et al., 2009), exacerbating ethanol‐induced hepatic damage (Marinello et al., 2019), and causing acute fulminant liver failure (Suga et al., 2021), perhaps by contributing to the formation of cytotoxic substances, such as formaldeyde and methylamine. However, whether dietary creatine affects biomarkers of liver health at the population level remains unknown. Therefore, the main aim of this study is to evaluate the association between creatine intake through regular diet and liver disease manifestations, including liver fibrosis, cirrhosis, and hepatic steatosis, among U.S. individuals aged 12 years and over, using open‐source data from the 2017–2018 National Health and Nutrition Examination Survey (NHANES).

## 2 METHODS

### 2.1 Study design

A cross‐sectional population‐based study.

### 2.2 Settings

The NHANES is a continual program of bi‐annual surveys designed to assess the health and nutritional status of noninstitutionalized adults and children in the United States. The program combines interviews (including demographic, socioeconomic, dietary, and health‐related questions), physical examinations (such as medical, dental, and physiological assessments), and laboratory tests. The NHANES is an extensive initiative of the National Center for Health Statistics (NCHS), a component of the United States Centers for Disease Control and Prevention; more details about the program are available elsewhere (NCHS, 2022). Data for this study were obtained from the NHANES 2017–2018 round through an open‐access database available at the United States Centers for Disease Control and Prevention/NCHS content source. The NHANES 2017–2018 round encompasses data collection from 30 different survey locations across the United States, with data collected through in‐home interviews and at mobile examination centers from January 2017 to December 2018.

### 2.3 Participants

The 2017–2018 NHANES round included a total of 9254 males and females aged >0 to 150 years. From the total sample population, we extracted data for participants who provided dietary data for individual foods via dietary interviews, examination data from liver ultrasound transient elastography (LUTE), and laboratory data on standard biochemistry profiles. All NHANES participants were eligible for two 24‐hour dietary recall interviews, and participants 12 years or older answered for themselves, with dietary interviewers conducted in‐person interviews in English and Spanish. However, only 1 year or older participants were eligible for the frequency of fish and shellfish consumption questions following the 24‐h recall. The suitable sample for standard biochemistry profiles included examined participants aged 12 years and older, while the eligible sample for LUTE included participants aged 12 years and over, nonpregnant, wearing no implanted electronic medical device or other mechanical impediments on the right side of the abdomen where measurements would be taken, and able to take a supine position for the examination. A final subsample for this study consisted of participants with at least one elastography variable available for the analyses. The ethical approval to conduct the NHANES 2017–2018 round was granted by the NHANES Institutional Review Board (Protocol #2018‐01, and continuation of Protocol #2011‐17), with the informed consent obtained from all participants.

### 2.4 Variables

The study outcomes (dependent variables) included LUTE indices: median liver stiffness (E), stiffness E interquartile range (IQRe), median controlled attenuated parameter (CAP), and CAP interquartile range (IQRc); and standard blood liver panel biomarkers (total bilirubin, alanine transaminase [ALT], aspartate transaminase [AST], AST‐to‐ALT ratio, alkaline phosphatase [ALP], gamma‐glutamyltransefase [GGT], and albumin). Diagnostic criteria for liver fibrosis and cirrhosis were E > 6 kPa and E > 11 kPa, respectively (Cho et al., 2021); hepatic steatosis was diagnosed with CAP > 268 dB/m (Fabrellas et al., 2018). The exposure (independent) variables were total dietary creatine intake and categories of creatine intake (see below). Potential confounders were gender, age at screening, family income to poverty ratio, body mass index, daily alcohol consumption, and total caloric intake.

### 2.5 Data sources and measurement

Dietary data were collected via two 24‐h dietary recall interviews. The first interview was collected in person in the mobile examination center, and the second interview was collected by telephone call 3–10 days later. Two types of dietary intake data were available for the 2017–2018 NHANES survey cycle, *Individual Foods* files and *Total Nutrient Intakes* files, as available from NHANES 2017–2018 dietary dataset. Individual Foods files contained detailed information about each food/beverage item (including the description, amount of, and nutrient content) reported by each participant through interviews, while Total Nutrient Intakes files contained a summary record of total energy and nutrient intakes from foods and beverages (NCHS, 2017). To calculate creatine intake, we first identified creatine‐containing foods (e.g., milk and milk products; meat, poultry, fish, and mixtures) using eight‐digit food codes from the U.S. Department of Agriculture (USDA) entries from Individual Foods files. We subsequently recorded the gram weight of each food component containing creatine (USDA codes from 11100000 to 28522000) and calculated the net intake of those foods for each individual by merging all relevant food items on a daily basis. Individual values for total grams of creatine consumed per day were computed using the average amount of creatine (e.g., 0.20 g/kg for milk‐based foods and 3.88 g/kg for meat‐based sources) across all creatine‐containing food sources. For this study, we calculated the mean creatine intakes of the two 24‐hour dietary recalls for each individual; the inputs did not include creatine obtained from nutritional supplements or pharmacological agents. Mean daily alcohol consumption and total caloric intake of the two dietary recalls for each individual were calculated from Total Nutrient Intakes files. LUTE provided objective measures for two liver disease manifestations, liver fibrosis and hepatic steatosis. A detailed description of the procedures was documented in the procedure manual of this component (NHANES, 2018). In short, LUTE measurements were obtained in the NHANES mobile examination center using an ultrasound device (FibroScan 502 V2 Touch, Echosens, Waltham, MA) equipped with a medium or extra‐large probe after a participant had fasted for at least 3 h. Liver fibrosis was measured by FibroScan device, which uses ultrasound and vibration‐controlled transient elastography to derive liver stiffness (E, expressed in kPa; the median of all valid measurements performed during the examination) and stiffness E interquartile range (IQRe, expressed in kPa and represents the interval around the median within which 50% of all valid measurements fall). The device also simultaneously measures the ultrasound attenuation related to the presence of hepatic steatosis and records CAP (expressed in dB/m; median of all valid measurements performed during the examination) and IQRc (expressed in dB/m, representing the interval around the median within which 50% of all valid measurements fall). The elastography examination was performed by NHANES health technicians, trained and certified by NHANES staff and the equipment manufacturer. Blood liver panel biomarkers were measured on a modular platform analyzer (Cobas 6000 C501, Roche Diagnostics, Indianapolis, IN) at mobile examination centers, with more detailed information about analytical methodologies, principles, and operating procedures available elsewhere (NHANES, 2017). In addition, the NHANES 2017–2018 Demographics and Examination Data components were explored to acquire information on the participants' general characteristics, including gender, age at screening, family income to poverty ratio, and body mass index.

### 2.6 Sources of bias

Potential selection bias has been addressed by using NHANES multistage sampling (see below) that increased the goal of having a representative sample. The NHANES design changes periodically to sample larger numbers of specific subgroups of particular public health interest to improve the reliability and precision of estimates of health status indicators for these population subgroups (NCHS, 2017). The oversampled subgroups in the 2017–2018 NHANES round included Hispanic individuals, non‐Hispanic black individuals, non‐Hispanic Asian individuals, non‐Hispanic white and other persons at or below 185% of the Department of Health and Human Services poverty guidelines, and non‐Hispanic white and other individuals aged 80 years and older (NCHS, 2017), with weighting applied when reporting summary results for the whole study sample. Information inaccuracies and bias have been addressed by adopting standardized and validated methods, and using objective measures whenever possible. Specifically, the ratio of the IQRe of liver stiffness to the median (IQRe/E) was calculated as an indicator of variability, with elastography results not reported if they had IQRe/E ≥30, as recommended by the elastography equipment manufacturer. Confounding has been addressed by using a list of other factors (e.g., gender, age at screening, annual income, body mass index, daily alcohol consumption, and total caloric intake) associated with relevant study outcomes.

### 2.7 Study size

The NHANES 2017–2018 survey examined a nationally representative sample of about 10,000 persons, with individuals located in counties across the country. The NHANES uses a complex, multistage probability sampling design to select the participants’ representative of the civilian, noninstitutionalized population residing in the 50 states and D.C. (NCHS, 2017). A final subsample for this study consisted of 5957 participants, with a detailed selection procedure explained previously.

### 2.8 Creatine intake categories

Besides using the mean daily intake of creatine as the primary exposure, we created three categories of creatine intake as the secondary exposure. These included the low‐intake group (<1.00 g/day), medium‐intake group (1.00–1.99 g/day), and high‐intake group (≥2.00 g/day), with the medium‐intake group excluded from the intergroup comparison, as described previously (Ostojic et al., 2021). This margin was chosen due to the fact that most individuals consume 1–2 g of dietary creatine per day, which is considered a recommended amount (Brosnan et al., 2011). We analyzed the liver disease manifestations occurrence across these two creatine‐intake categories.

### 2.9 Statistical methods

A descriptive analysis comparing demographic and nutritional variables across groups of dietary creatine intake was conducted using adjusted chi‐square tests and independent *t* tests. Linear regression was used to assess the association between dietary creatine intake and liver panel outcomes, while the bivariate logistic regression was conducted to determine the association between subsamples with low‐intake and high‐intake creatine consumption and liver condition; regression models were adjusted for an a priori defined set of covariates. Data were analyzed using SPSS Statistics for Mac (Version 24.0; IBM), with the significance level set at *p* < .05.

## 3 RESULTS

The number of individuals from the NHANES 2017–2018 round whole sample was 9254, with 7487 participants providing information on dietary intake of creatine. Of those, at least one LUTE outcome was available for 5957 individuals (age, 44.7 ± 21.0 years, 2987 [50.1%] female) who were included in the final analyses. The demographic and clinical characteristics of the study participants are depicted in Table 1. The mean ± SD dietary creatine intake across the study population was 0.88 ± 0.71 g/day. The high‐intake creatine consumers (≥2 g of creatine per day) were more likely to be men, had higher alcohol intake, higher total caloric content, and different several liver panel indices (including ALT, AST‐to‐ALT ratio, ALP, and albumin) compared to the low‐intake group (<1 g of creatine per day).

**TABLE 1:** Characteristics of the study population

	Creatine intake			
Characteristic	Low intake (<1 g/day) (*n* = 3546)	Medium intake (1–1.99 g/day) (*n* = 1458)	High intake (≥2.00 g/day) (*n* = 364)	p*
Age (years), mean (SD)	44.5 (21.4)	45.0 (20.1)	43.7 (18.1)	.46
Sex (%)				
Male	42.2	62.1	77.7	<.001
Female	57.8	37.9	22.3	
Family income to poverty ratio	2.5 (1.6)	2.5 (1.6)	2.4 (1.6)	.30
BMI (kg/m^2^), mean	28.7 (7.3)	29.5 (7.9)	28.8 (7.3)	.78
Dietary data				
Creatine intake (g/day), mean	0.50 (0.28)	1.36 (0.26)	2.68 (0.86)	<.001
Alcohol intake (g/day), mean (SE)	5.3 (0.3)	8.3 (0.6)	10.3 (1.7)	<.001
Total energy intake (kcal/day), mean (SE)	1819 (12)	2317 (22)	2928 (65)	<.001
LUTE outcomes				
Median E (kPa), mean	5.8 (5.0)	6.2 (5.5)	6.1 (5.5)	.36
IQRe, mean	0.98 (2.11)	1.04 (2.33)	1.03 (2.47)	.70
IQRe/median E, mean	15.7 (25.2)	15.0 (10.1)	14.7 (8.7)	.45
Median CAP (dB/m), mean	257.5 (62.9)	262.6 (66.2)	258.2 (65.6)	.83
IQRc, mean	38.6 (20.7)	38.1 (21.2)	38.9 (21.9)	.74
Standard liver panel				
Total bilirubin (μmol/L), mean	7.8 (4.8)	8.0 (4.9)	8.1 (4.2)	.28
AST (IU/L), mean	22 (14)	22 (11)	22 (9)	.49
ALT (IU/L), mean	21 (18)	23 (16)	25 (17)	<.001
AST‐to‐ALT ratio	1.21 (0.47)	1.13 (0.43)	1.02 (0.35)	<.001
ALP (IU/L), mean	93 (56)	90 (50)	80 (34)	<.001
GGT (IU/L), mean	29 (45)	31 (40)	32 (36)	.17
Albumin (g/dl), mean	4.0 (0.3)	4.1 (0.3)	4.1 (0.3)	.003

*Note*: *p for comparison between low‐intake and high‐intake groups, with *p* < .05 considered statistically significant.

Abbreviations: ALP, alkaline phosphatase; ALT, alanine transaminase; AST, aspartate transaminase; BMI, body mass index (calculated as weight in kilograms divided by height in meters squared). GGT, gamma‐glutamyltransefase; IQRc, CAP interquartile; IQRe, stiffness E interquartile range; LUTE, liver ultrasound transient elastography; median CAP, median controlled attenuated parameter; median E, median liver stiffness.

**FIGURE 1:**
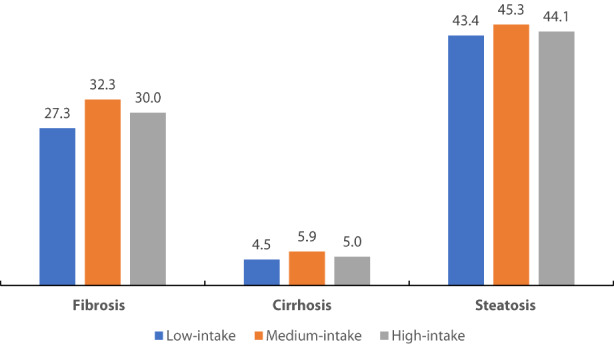
The occurrence (%) of liver manifestations across different groups of creatine intake. No differences found between low‐intake (<1 g/day) and high‐intake (≥2 g/day) consumers (*p* > .05).

Liver fibrosis and cirrhosis were diagnosed in 1703 (28.7%) and 288 (4.8%) participants, respectively; hepatic steatosis was identified in 2595 (43.7%) individuals. No differences were found between the occurrence of liver fibrosis, cirrhosis, and hepatic steatosis between individuals consuming low and high amounts of creatine via regular diet (*p* > .05) (Figure 1). Median E and CAP correlated weakly with mean daily creatine intake (*r* = .028, *p* = .04 and *r* = .032, *p* = .02, respectively), while no significant correlation was reported between creatine intake and other LUTE outcomes (*p* > .05). We also found weak correlations between most liver panel variables and creatine intake (total bilirubin: *r* = .03, *p* = .04; ALT: *r* = .08, *p* < .001; AST‐to‐ALT ratio: *r* = −.12, *p* < .001; ALP: *r* = −.06, *p*< .001; GGT: *r* = .03, *p* = .02; and albumin: *r* = .04, *p* = .003).

**FIGURE 2:**
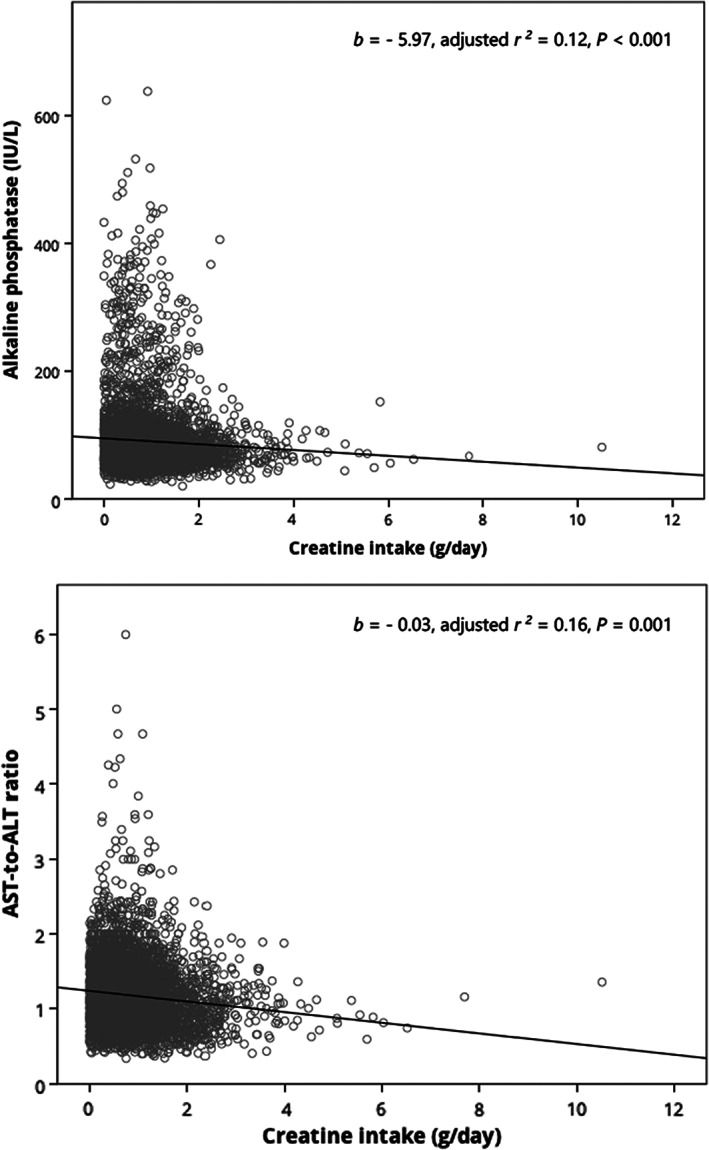
Regression models with significant association (*p* < .05) between dietary creatine intake and liver panel outcomes in U.S. individuals aged 12 years and over.

The results of linear regression adjusted for cofounding variables displayed no significant link between creatine intake and most lipid panel variables (albumin, *b* = 0.01, *p* = .32; ALT, *b* = 0.26, *p* = .50; AST, *b* = −0.48, *p* = .09; GGT, *b* = −0.59, *p* = .54; total bilirubin, *b* = −0.06, *p* = .60), except for ALP (*b* = −5.97, *p* < .001) and AST‐to‐ALT ratio (*b* = −0.03, *p* = .001) (Figure 2). In addition, binary logistic regression with multivariable model adjusted for age, gender, family income to poverty ratio, body mass index, total energy intake, and alcohol consumption showed that consuming more creatine (≥2 g/day) did not significantly increase the risk of liver fibrosis (OR = 0.92, 95% CI 0.70–1.21, *p* = .57), cirrhosis (OR = 0.94, 95% CI 0.53–1.65, *p* = .82), and hepatic steatosis (OR = 0.77, 95% CI 0.59–1.02, *p* = .07), as compared to participants who ingested <1 g of creatine daily.

## 4 DISCUSSION

To our knowledge, this is the first population‐based study that evaluated the association between creatine intake through regular diet and liver disease manifestations in the general public aged 12 years and older. We found no differences between the occurrence of liver fibrosis, cirrhosis, and hepatic steatosis between the individuals taking <1 g of creatine per day (low‐intake consumers) and those who consume over 2 g/day of creatine via regular diet (high‐intake consumers), with the risk of having liver conditions similar between two opposite groups of creatine consumption. Our findings indicate no link between biomarkers of liver damage and additional food‐driven creatine in this population. Still, further research is warranted to address the possible effects of excessive creatine intake (e.g., >5 g/day) through a regular diet on liver health at the population level.

About a dozen safety studies about the possible hepatotoxic effects of dietary creatine are available in the scientific literature. Most trials demonstrated no harmful impact of creatine intake on liver functions, including no disturbances in liver enzymes, hepatic histopathology, and organ‐specific clinical outcomes (Mayhew et al., 2002; Kreider et al., 2003; Ramos Fernandes et al., 2022), with some reporting hepatoprotective effects of creatine consumption (Araújo et al., 2013; da Silva et al., 2014; Deminice et al., 2015). However, several experimental studies found possible adverse effects of exogenous creatine when consumed excessively. Souza et al. (2009) found that rodents consuming higher doses of dietary creatine experienced a significant rise in liver enzymes panel, accompanied by structural alterations indicating hepatic damage. These findings were corroborated in another preclinical study where creatine provoked an increase in several hepatic biomarkers (Souza et al., 2013). In another study, ethanol intake combined with creatine exacerbated cell degeneration and fat accumulation, hepatic expression of genes related to ethanol metabolism, oxidative stress, and inflammation, and promoted oxidative stress and elevated plasma alanine aminotransferase (Marinello et al., 2019). In addition, several case reports demonstrated a high‐dose creatine‐mediated liver injury in young men who took creatine (but also other nutritional products) as an aid for exercise training (Avelar‐Escobar et al., 2012; Suga et al., 2021; Whitt et al., 2008), further raising concerns about possible adverse effects of creatine when administered above‐recommended amounts. These conflicting results might be due to the variability of creatine dosages used, the duration of the intervention, the species evaluated (e.g., rodents vs. humans), concomitant conditions present, and/or coadministration of other compounds (e.g., alcohol, protein, nitrates), with above studies typically low powered and limited in methods used to evaluate liver function.

Our current cross‐sectional study expands the previous research to a population‐wide sample while using standardized methods to evaluate liver health and controlling for critical confounding variables, confirming no significant links between creatine consumption and liver dysfunction within the context of a regular diet. We found no differences in risks from liver malfunctions (e.g., fibrosis, cirrhosis, and steatosis) between individuals consuming a low amount of creatine (<1 g/day) and those taking over 2 g of creatine per day in U.S. individuals aged 12 years and over. Consuming over fivefold more creatine (mean intake of 2.68 g/day for the high‐intake group) seemed not to be associated with more liver disease manifestations than low‐intake consumption (mean intake of 0.50 g/day). In addition, taking more creatine might be related to lower ALP levels and AST‐to‐ALT ratio, perhaps suggesting hepatoprotective effects of additional creatine from the regular diet. In line with this, we also found that for each additional gram of creatine consumed per day, the expected reductions in liver median stiffness (an indicator of liver fibrosis) and median controlled attenuated parameter (an indicator of hepatic steatosis) were 0.04 kPa and 2.22 dB/m, respectively. Our data extend recent population‐based findings, where additional creatine was associated with a lower risk of liver conditions in the elderly (Ostojic et al., 2021). The previous study obtained data on liver conditions through in‐person interviews, while our study used a gold‐standard method to evaluate chronic liver conditions at the population level (Mumtaz et al., 2019).

The present cross‐sectional study is not without limitations. Our design restricts any claims about the cause–effect relationship; dietary data collection used here is prone to participants recall bias and poorly reflects a typical diet; the creatine calculation method uses an equivalent amount of creatine across all relevant foods, while this may vary slightly in different meat products (Purchas et al., 2004), and no objective indicators of creatine status have been monitored while not considering endogenous creatine yield. In addition, we used mainly biomarkers characterizing chronic liver diseases which partly limit any conclusions about dietary creatine and acute liver conditions. A possible contribution of hepatotoxic medications consumed has not been accounted for the outcome variables analyzed. Likewise, dietary intake of other nutrients relevant for liver health (e.g., sodium, saturated fats, total sugars) in creatine‐containing foods could also affect the relationship between creatine consumption and liver disease manifestations, requiring further investigation.

Chronic liver conditions are significant contributors to the morbidity in U.S. population during the past three decades (Younossi et al., 2020), with the prevalence of several chronic diseases (such as nonalcoholic hepatic steatosis) continues to grow. Poor diet not only is a potential factor in the pathogenesis of nonalcoholic hepatic steatosis, but also plays an important role in its treatment (Hernandez‐Rodas et al., 2015). Therefore, the recognition of single nutrients or dietary patterns associated with chronic liver diseases remains of utmost importance. Our study found no strong link between a diet rich in creatine‐containing foods and the higher risk of liver fibrosis and steatosis, suggesting this nutritional compound might not be of major relevance for chronic liver conditions in the general population. However, a mild reduction of liver fibrosis/steatosis indices with additional creatine consumed reported in this study could be of interest, but more studies are required to clarify this potentially favorable interrelationship.

## 5 CONCLUSION

Dietary exposure to creatine through a regular diet is not associated with more liver disease manifestations in U.S. population aged 12 years and over. The risk of having liver fibrosis, cirrhosis, and hepatic steatosis is similar between low‐intake and high‐intake creatine consumers. In addition, taking creatine from food sources might be associated with favorable individual liver function tests; further safety studies are needed to address the upper threshold for dietary creatine intake in the general public.

## FUNDING INFORMATION

None received.

## CONFLICT OF INTEREST

SMO serves as a member of the Scientific Advisory Board on creatine in health and medicine (AlzChem LLC). SMO owns patent “Supplements Based on Liquid Creatine” at the European Patent Office (WO2019150323 A1), and patent “Methods and Compositions for Improving a Response to a Metabolic Stress” at the United States Patent and Trademark Office (U.S. 2015/0150933 A1). SMO has served as a speaker at Abbott Nutrition and has received research funding related to creatine during the past 36 months from the Serbian Ministry of Education, Science, and Technological Development, the Provincial Secretariat for Higher Education and Scientific Research, AlzChem GmbH, ThermoLife International, and Hueston Hennigan LLP. SMO does not own stocks and shares in any organization. DK and VS declare no conflict of interest.

## ETHICS STATEMENT

The study conforms to the Declaration of Helsinki, U.S., and European Medicines Agency Guidelines for human subjects. The study's protocols and procedures were ethically reviewed and approved by the NHANES Institutional Review Board (Protocol #2018‐01, effective beginning October 26, 2017; and continuation of Protocol #2011‐17, effective through October 26, 2017), with the informed consent obtained from all participants.

## Data Availability

Data described in the manuscript will be made publicly and freely available without restriction upon request.

## References

[fsn33151-bib-0001] Araújo, M. B. , Moura, L. P. , Junior, R. C. , Junior, M. C. , Dalia, R. A. , Sponton, A. C. , Ribeiro, C. , & Mello, M. A. (2013). Creatine supplementation and oxidative stress in rat liver. Journal of the International Society of Sports Nutrition, 10(1), 54. 10.1186/1550-2783-10-54 24325803 PMC4029397

[fsn33151-bib-0002] Avelar‐Escobar, G. , Méndez‐Navarro, J. , Ortiz‐Olvera, N. X. , Castellanos, G. , Ramos, R. , Gallardo‐Cabrera, V. E. , Vargas‐Alemán Jde, J. , Díaz de León, O. , Rodríguez, E. V. , & Dehesa‐Violante, M. (2012). Hepatotoxicity associated with dietary energy supplements: Use and abuse by young athletes. Annals of Hepatology, 11(4), 564–569. 22700641

[fsn33151-bib-0003] Brosnan, J. T. , da Silva, R. P. , & Brosnan, M. E. (2011). The metabolic burden of creatine synthesis. Amino Acids, 40(5), 1325–1331. 10.1007/s00726-011-0853-y 21387089

[fsn33151-bib-0004] Cho, Y. , Kabata, D. , Ehara, E. , Yamamoto, A. , Mizuochi, T. , Mushiake, S. , Kusano, H. , Kuwae, Y. , Suzuki, T. , Uchida‐Kobayashi, S. , Morikawa, H. , Amano‐Teranishi, Y. , Kioka, K. , Jogo, A. , Isoura, Y. , Hamazaki, T. , Murakami, Y. , & Tokuhara, D. (2021). Assessing liver stiffness with conventional cut‐off values overestimates liver fibrosis staging in patients who received the Fontan procedure. Hepatology Research, 51(5), 593–602. 10.1111/hepr.13627 33677839

[fsn33151-bib-0005] da Silva, R. P. , Kelly, K. B. , Leonard, K. A. , & Jacobs, R. L. (2014). Creatine reduces hepatic T.G. accumulation in hepatocytes by stimulating fatty acid oxidation. Biochimica et Biophysica Acta, 1841(11), 1639–1646. 10.1016/j.bbalip.2014.09.001 25205520

[fsn33151-bib-0006] Deminice, R. , de Castro, G. S. , Francisco, L. V. , da Silva, L. E. , Cardoso, J. F. , Frajacomo, F. T. , Teodoro, B. G. , Dos Reis, S. L. , & Jordao, A. A. (2015). Creatine supplementation prevents fatty liver in rats fed choline‐deficient diet: A burden of one‐carbon and fatty acid metabolism. The Journal of Nutritional Biochemistry, 26(4), 391–397. 10.1016/j.jnutbio.2014.11.014 25649792

[fsn33151-bib-0007] Fabrellas, N. , Hernández, R. , Graupera, I. , Solà, E. , Ramos, P. , Martín, N. , Sáez, G. , Simón, C. , Pérez, A. , Graell, T. , Larrañaga, A. , Garcia, M. , de la Arada, A. , Juanola, A. , Coiduras, A. , Duaso, I. , Casado, A. , Martin, J. , Ginès, M. , … Ginès, P. (2018). Prevalence of hepatic steatosis as assessed by controlled attenuation parameter (CAP) in subjects with metabolic risk factors in primary care. A population‐based study. PLoS One, 13(9), e0200656. 10.1371/journal.pone.0200656 30226889 PMC6143232

[fsn33151-bib-0008] Hernandez‐Rodas, M. C. , Valenzuela, R. , & Videla, L. A. (2015). Relevant aspects of nutritional and dietary interventions in non‐alcoholic fatty liver disease. International Journal of Molecular Sciences, 16(10), 25168–25198. 10.3390/ijms161025168 26512643 PMC4632797

[fsn33151-bib-0009] Kreider, R. B. , Jäger, R. , & Purpura, M. (2022). Bioavailability, efficacy, safety, and regulatory status of creatine and related compounds: A critical review. Nutrients, 14(5), 1035. 10.3390/nu14051035 35268011 PMC8912867

[fsn33151-bib-0010] Kreider, R. B. , Melton, C. , Rasmussen, C. J. , Greenwood, M. , Lancaster, S. , Cantler, E. C. , Milnor, P. , & Almada, A. L. (2003). Long‐term creatine supplementation does not significantly affect clinical markers of health in athletes. Molecular and Cellular Biochemistry, 244(1–2), 95–104. 12701816

[fsn33151-bib-0011] Marinello, P. C. , Cella, P. S. , Testa, M. T. J. , Guirro, P. B. , Brito, W. A. S. , Borges, F. H. , Cecchini, R. , Cecchini, A. L. , Duarte, J. A. , & Deminice, R. (2019). Creatine supplementation exacerbates ethanol‐induced hepatic damage in mice. Nutrition, 66, 122–130. 10.1016/j.nut.2019.05.004 31265967

[fsn33151-bib-0012] Mayhew, D. L. , Mayhew, J. L. , & Ware, J. S. (2002). Effects of long‐term creatine supplementation on liver and kidney functions in American college football players. International Journal of Sport Nutrition and Exercise Metabolism, 12(4), 453–460. 10.1123/ijsnem.12.4.453 12500988

[fsn33151-bib-0013] Mumtaz, S. , Schomaker, N. , & Von Roenn, N. (2019). Pro: Noninvasive imaging has replaced biopsy as the gold standard in the evaluation of nonalcoholic fatty liver disease. Clinical Liver Disease (Hoboken)., 13(4), 111–113. 10.1002/cld.750 PMC6491026 31061704

[fsn33151-bib-0014] NHANES (National Health and Nutrition Examination Survey) . (2018). Liver ultrasound transient elastography procedures manual. Centers for Disease and Prevention.

[fsn33151-bib-0015] NHANES (National Health and Nutrition Examination Survey) . (2017). MEC laboratory procedures manual. Centers for Disease and Prevention.

[fsn33151-bib-0016] NCHS (National Center for Health Statistics) . (2017). About the National Health and Nutrition Examination Survey https://www.cdc.gov/nchs/nhanes/about_nhanes.htm Page last reviewed: September 15, 2017. Assessed at August 11, 2022.

[fsn33151-bib-0017] NCHS (National Center for Health Statistics) . (2022). NHANES 2017–2018 Dietary Data https://wwwn.cdc.gov/nchs/nhanes/search/datapage.aspx?Component=Dietary&Cycle=2017‐2018 Assessed at August 11 2022.

[fsn33151-bib-0018] Ostojic, S. M. , & Forbes, S. C. (2022). Perspective: Creatine, a conditionally essential nutrient: Building the case. Advances in Nutrition, 13(1), 34–37. 10.1093/advances/nmab111 34662902 PMC8803499

[fsn33151-bib-0019] Ostojic, S. M. , Korovljev, D. , & Stajer, V. (2021). Dietary intake of creatine and risk of medical conditions in U.S. older men and women: Data from the 2017‐2018 National Health and nutrition examination survey. Food Science & Nutrition, 9(10), 5746–5754. 10.1002/fsn3.2543 34646542 PMC8498075

[fsn33151-bib-0020] Purchas, R. W. , Rutherfurd, S. M. , Pearce, P. D. , Vather, R. , & Wilkinson, B. H. (2004). Concentrations in beef and lamb of taurine, carnosine, coenzyme Q(10), and creatine. Meat Science, 66(3), 629–637. 10.1016/S0309-1740(03)00181-5 22060873

[fsn33151-bib-0021] Ramos Fernandes, V. A. , Delforno, M. C. , Banov, G. C. , Shmayev, M. , Alves Leandro, J. V. , Gonçalves Teixeira, K. F. , Iatecola, A. , Inácio Cardozo, M. F. , Caldeira, E. J. , & Rodrigues da Cunha, M. (2022). Renal, hepatic and muscle effects of creatine supplementation in an older adults experimental model. Clinical Nutrition ESPEN, 48, 464–471. 10.1016/j.clnesp.2021.12.020 35331530

[fsn33151-bib-0022] Riesberg, L. A. , Weed, S. A. , McDonald, T. L. , Eckerson, J. M. , & Drescher, K. M. (2016). Beyond muscles: The untapped potential of creatine. International Immunopharmacology, 37, 31–42. 10.1016/j.intimp.2015.12.034 26778152 PMC4915971

[fsn33151-bib-0023] Souza, R. A. , Miranda, H. , Xavier, M. , Lazo‐Osorio, R. A. , Gouvea, H. A. , Cogo, J. C. , Vieira, R. P. , & Ribeiro, W. (2009). Effects of high‐dose creatine supplementation on kidney and liver responses in sedentary and exercised rats. Journal of Sports Science and Medicine, 8(4), 672–681. 24149610 PMC3761536

[fsn33151-bib-0024] Souza, W. M. , Heck, T. G. , Wronski, E. C. , Ulbrich, A. Z. , & Boff, E. (2013). Effects of creatine supplementation on biomarkers of hepatic and renal function in young trained rats. Toxicology Mechanisms and Methods, 23(9), 697–701. 10.3109/15376516.2013.843108 24024661

[fsn33151-bib-0025] Suga, S. H. , Dasu, N. R. , Khalid, Y. , Dasu, K. B. S. , Elgenaidi, H. , & McMahon, D. J. (2021). A rare case of acute fulminant liver failure caused by body building supplements. American Journal of Gastroenterology., 116, S1205. 10.14309/01.ajg.0000785168.79760.c1

[fsn33151-bib-0026] Younossi, Z. M. , Stepanova, M. , Younossi, Y. , Golabi, P. , Mishra, A. , Rafiq, N. , & Henry, L. (2020). Epidemiology of chronic liver diseases in the USA in the past three decades. Gut, 69(3), 564–568. 10.1136/gutjnl-2019-318813 31366455

[fsn33151-bib-0027] Whitt, K. N. , Ward, S. C. , Deniz, K. , Liu, L. , Odin, J. A. , & Qin, L. (2008). Cholestatic liver injury associated with whey protein and creatine supplements. Seminars in Liver Disease, 28(2), 226–231. 10.1055/s-2008-1073122 18452122

[fsn33151-bib-0028] Wyss, M. , & Kaddurah‐Daouk, R. (2000). Creatine and creatinine metabolism. Physiological Reviews, 80(3), 1107–1213. 10.1152/physrev.2000.80.3.1107 10893433

